# Dose-Effects Models for Space Radiobiology: An Overview on Dose-Effect Relationships

**DOI:** 10.3389/fpubh.2021.733337

**Published:** 2021-11-08

**Authors:** Lidia Strigari, Silvia Strolin, Alessio Giuseppe Morganti, Alessandro Bartoloni

**Affiliations:** ^1^Department of Medical Physics, Istituto di Ricovero e Cura a Carattere Scientifico (IRCCS) Azienda Ospedaliero-Universitaria di Bologna, Bologna, Italy; ^2^Radiation Oncology Center, School of Medicine, Department of Experimental, Diagnostic and Specialty Medicine - DIMES, University of Bologna, Bologna, Italy; ^3^Istituto Nazionale di Fisica Nucleare (INFN) Sezione di Roma 1, Roma, Italy

**Keywords:** human space exploration, galactic cosmic radiation, galactic cosmic radiation effects, space radiobiology, space radiation doses, dose-effect model

## Abstract

Space radiobiology is an interdisciplinary science that examines the biological effects of ionizing radiation on humans involved in aerospace missions. The dose-effect models are one of the relevant topics of space radiobiology. Their knowledge is crucial for optimizing radioprotection strategies (e.g., spaceship and lunar space station-shielding and lunar/Mars village design), the risk assessment of the health hazard related to human space exploration, and reducing damages induced to astronauts from galactic cosmic radiation. Dose-effect relationships describe the observed damages to normal tissues or cancer induction during and after space flights. They are developed for the various dose ranges and radiation qualities characterizing the actual and the forecast space missions [International Space Station (ISS) and solar system exploration]. Based on a Pubmed search including 53 papers reporting the collected dose-effect relationships after space missions or in ground simulations, 7 significant dose-effect relationships (e.g., eye flashes, cataract, central nervous systems, cardiovascular disease, cancer, chromosomal aberrations, and biomarkers) have been identified. For each considered effect, the absorbed dose thresholds and the uncertainties/limitations of the developed relationships are summarized and discussed. The current knowledge on this topic can benefit from further *in vitro* and *in vivo* radiobiological studies, an accurate characterization of the quality of space radiation, and the numerous experimental dose-effects data derived from the experience in the clinical use of ionizing radiation for diagnostic or treatments with doses similar to those foreseen for the future space missions. The growing number of pooled studies could improve the prediction ability of dose-effect relationships for space exposure and reduce their uncertainty level. Novel research in the field is of paramount importance to reduce damages to astronauts from cosmic radiation before Beyond Low Earth Orbit exploration in the next future. The study aims at providing an overview of the published dose-effect relationships and illustrates novel perspectives to inspire future research.

## Introduction

Space radiobiology (SPRB) is a fascinating field that has fostered a growing interest in the recent years, thanks to the increased technological capability to travel and operate in space and the consequent renewed interest from the national space agencies to plan exploratory and colonization space missions.

**Figure 1:**
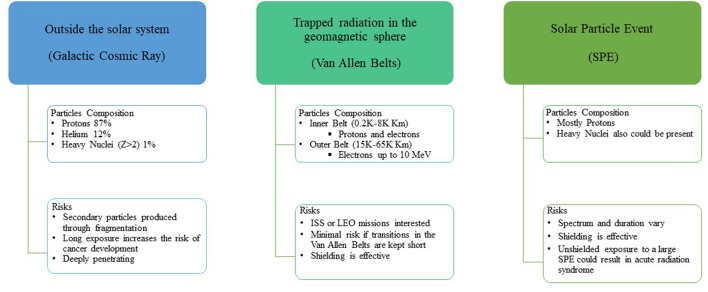
Scheme of the origin of space radiation particles and consequent risk. ISS, International Space Station; LEO, Low Earth Orbit.

The space radiation environment is a complex mixture of radiation species dominated by highly penetrating charged particles from different sources (Figure 1). In this regard, three different sources of particles are present: particles emitted by the Sun (SPE) due to the solar activities, particles trapped in the magnetic field of the Earth (i.e., Radiation Belt), and galactic cosmic rays (GCRs) coming from outside the solar system.

Planetary magnetic fields and short-term and long-term solar activities modulate the energy spectrum and abundances of radiation species (1). In addition, the presence of shielding on the space stations or spacecraft modifies the incident spectrum and related exposure due to (secondary) particles production resulting from the interaction (spallation) of the space radiation particles with such structures.

Such particles can penetrate several hundreds of centimeters of materials, such as aluminum or tissue/water and fragment before stopping, producing lower Z secondary particles through nuclear interactions. A lower linear energy transfer (LET) characterizes the secondary particles. Such characteristics confer a higher penetration range than the primary particles (2).

Ionizing radiation protection on Earth uses several technical solutions to reduce the exposure of workers: increasing the distance from the radiation source, reducing the exposure time, and implementing *ad hoc* shielding (3).

Distance is not helpful in space since GCRs are mostly isotropically distributed. Time exposure reduction is a valid approach in space, but not practical due to spacecraft velocity or time to perform scientific tasks. Further, it will be significantly longer than what has been experienced so far for the planned exploration and colonization to Moon and Mars. Shielding, either active or passive, is crucial to reduce radiation exposure significantly but cannot fully absorb all space radiation due to the high-energy component and the time-variable contribution of the GCR spectrum. In addition, shielding materials and thickness need to be optimized considering their efficacy and cost to reduce the unavoidable exposures to the minimum acceptable level. Nevertheless, dose- and equivalent dose-rate of the astronauts are around 0.3–0.6 mGy/day, corresponding to 1–1.8 mSv/day, respectively (4).

Both acute and late effects in the space radiation environment are the most frequent and relevant life-threatening adverse events associated with ionizing radiation exposure. Acute radiation syndrome (i.e., short-term effects) is caused by intense and short exposure to SPEs in case of crews unable to reach areas with adequate shielding. Late radiation morbidity [e.g., carcinogenesis or central nervous system (CNS) damage] is associated with continuous exposure to GCR, which is substantially different both qualitatively and quantitatively from the natural background of the radiation of Earth, depending on various above-described factors (i.e., long- or short-term solar activity and magnetic field features).

Mathematical models of dose-effect relationships are developed and are confirmed not only from human studies but also from *in vitro* cell or *in vivo* small animal studies. Such models explain and predict the clinical and subclinical effects recorded during space missions. In addition, it is possible to use clinical diagnostic or radiotherapeutic devices for performing a ground simulation of GCR scenarios and improving the space exposure radiobiological model understanding, due to the similarity of dosage and type of available particles. Moreover, the complete understanding of non-targeted effects induced by charged particles becomes mandatory (5) due to the interaction of secondary particles with several human healthy tissues. Nontargeted effects may dominate cancer risk at space-relevant doses. Furthermore, several investigations are still ongoing to consider the possibility of hibernating astronauts to guarantee additional protection against space radiation effects, given the radioprotective action of hypothermia (6).

Our study aims at reviewing the acute and late adverse effects of space travel to be compared/discussed to the ones currently observed after diagnostic or radiotherapy exposure or through ground simulations to similar dosage/radiations.

## Materials and Methods

### Resource Identification Search Strategy

The performed PubMed search uses the query string reported below to identify the proposed models for acute and late effects related to space mission/exposure and compare these effects with the threshold reported in the diagnostic or therapeutic applications using ionizing radiation. Query search included the following keywords/string: space[title/abstract] model[title/abstract] radiobiol^*^ [title/abstract]. For each detrimental health and tissue effect, an additional search have to be implemented e.g., (model[title/abstract] OR relationship[title/abstract]) AND (radiotherapy[title/abstract] OR space[title/abstract]) AND (radiobiol^*^ [title/abstract] OR dose [title/abstract]).

The research had been restricted to the last 10 years to include only the most recently published studies. The last search was done on August 30, 2021. The authors independently reviewed titles and abstracts to decide study inclusion. Full articles were retrieved when the abstract was considered relevant, and only papers published in English were considered. The bibliographies of retrieved and reviewed papers were also examined to identify other relevant articles to be included and published before 2011. Papers were considered eligible when reporting models and dose-effect correlations.

Analyzed effects included, among others, eye flashes, cataracts, CNS effects, cardiovascular disease (CVD), biomarkers including chromosomal aberrations, cancer induction (including mortality), and other possible risks never evidenced in astronauts but investigated as possible long-term irradiation for future missions to Mars and Moon (Table 1). In the last columns of Table 1, the overall reliability and research priority rates are reported using a 5-point scoring system from very low (^*^) to very high (^*****^) values. The reliability of models has been reported considering the number of revealed effects, statistical approaches, and information on dose and GCR spectrum and its modification through shielding materials. We also include an attempt to score the priority for future research considering the possible impact on a long-term mission in deep space, the availability of advanced facilities, and the possible synergies with related medical fields using ionizing radiation.

**Table 1:** Dose-effect relationship for space radiation risk assessment.

**Model**	**Study type**	**Dose range/threshold or LET**	**Reference**	**Reliability**	**Priority**
Eye flashes	Spaceflight	LET> 5–10 keV/μm	(7–10)	^****^	^*^
Cataract	Spaceflight	8 mSv	(11–15)	^***^	^***^
CNS	Ground/Simulation	100–200 mGy	(16–27)	^**^	^*****^
CVD	Spaceflight	1000 mGy	(28–31)	^*^	^***^
	Ground/Simulation	(0.1–4,500) mSv	(32–39)		
Cancer	Spaceflight	<100 mGy	(40, 41)	^***^	^*****^
	Ground/Simulation	<100 mGy	(42–50)		
Biomarkers or	Spaceflight	5–150 mGy	(51–61)	^***^	^*****^
Chromosomal aberrations	Ground/Simulation	<10,000 mGy	(62–65)		
Other Risks	Ground/Simulation	~2,000 mGy	(66, 67)	^*^	^***^

* *= Very Low*,

** *= Low*,

*** *= Medium*,

**** *= High*,

***** *= Very High*.

## Results

### Identified Studies

Based on Pubmed/Medline search, 61 papers have been found. Among this, 54 were original papers reporting/proposing radiobiology or dose-effects models, while 8 were reviews or relevant reports (which were screened for including additional papers). About 24 papers mainly focused on data obtained from astronauts or spaceflight crews, while 37 were generated using ground experiments and/or simulations. Other reports or commentary papers were included in the discussion.

### Dose-Effect Relationships

The identified models based on available data from spaceflight missions or ground/simulation data have been described in Table 1 and more in detail in the subsequent paragraphs. The scores of overall reliability and research priority are also reported in Table 1. The scores of overall reliability and research priority are associated with the robustness of identified models and expected doses calculated for long-term missions considering that astronauts are healthy non-smoker subjects. The higher priorities regard cancer, biomarker/sensitivity, and CNS risk, which can potentially affect the duration and quality of life of space crew/astronauts. Regardless of the relevant efforts in the last decades, the reliability of models is still sub-optimal for most of the medium- and long-term effects.

The effects of radiation exposure in space can manifest themselves at different times; short-term (e.g., eye flashes) are observed immediately and are transient, medium-term [e.g., CVD] after several weeks/months depending on the absorbed dose, and long-term (e.g., cancer) can also occur many years (10 to 30) after exposure. The performance, characteristics, limitations, and uncertainties of these dose-effect models are discussed in the following paragraphs.

**Figure 2:**
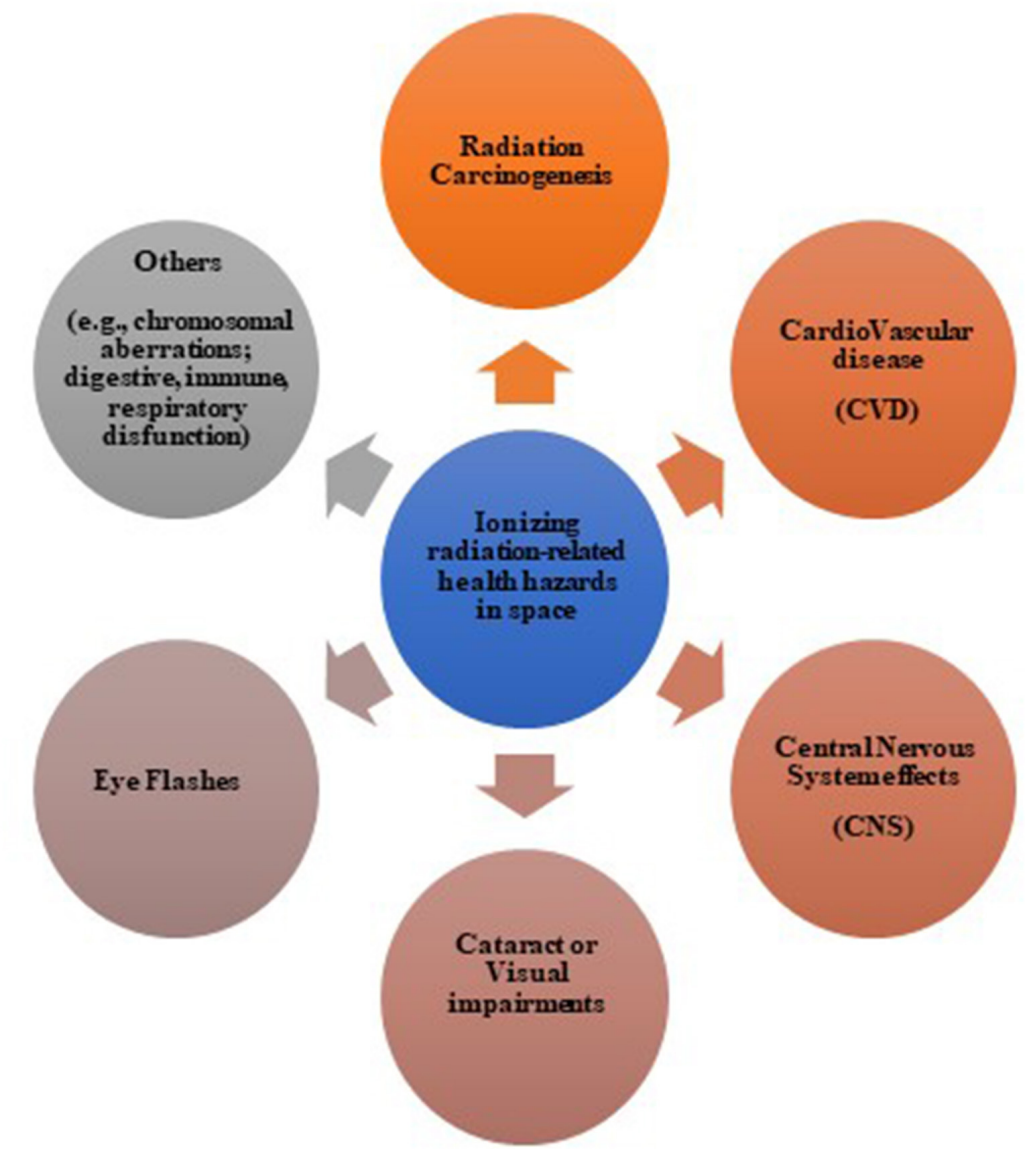
Possible ionizing radiation-related health hazards in space.

Figure 2 shows the several possible ionizing radiation-induced effects during and after space missions, including acute and late effects on normal tissues, as well as neurological disease and lens opacification, and cancer risk. Figure 2 also includes the oral mucositis and CVD, which have never registered at the doses absorbed by astronauts until now but represent a potential risk in case of prolonged exposure as expected after the Mars colonization.

### Eye Flashes

#### Spaceflight-Based Studies

More in detail, the first description of the biological consequences of the space radiation exposure on human cells was the subjective sensations of lights on eyes, commonly called eye flashes, first observed by Apollo crews (7). The origin of the eye flashes remained unresolved for several years. Geometrical considerations and the Monte Carlo calculations show that, at least in part, the flashes seen by astronauts are correlated with charged particles traversing the retina. Primary or secondary neutrons and possibly heavy ions, rather than mesons, were suspected to cause eye flashes. Observations on the helmets of Apollo astronauts reveal numerous tracks of metallic ions as heavy as zinc and nickel, which are very rare in space, suggesting that the technical environment of spacecraft itself adds extra complexity to the actual spectrum of secondary particles. Secondary particles, generated by the interaction of very high-energy particles with metallic stuff of spacecraft, having the a LET >5–10 keV/μm were suspected to cause eye flashes (8, 9). The phenomenon of light flashes in space investigated onboard Mir space station correlated the data on particles hitting the eye collected with the SilEye detectors with human observations. Likely, a nucleus in the radiation environment of the Mir has roughly a 1% probability of causing eye flashes, whereas the proton probability is almost of three orders of lesser magnitude. The probability of the eye flashes increases above 10 keV/micrometer as a LET function, reaching about 5% at around 50 keV/micrometer (9). Preliminary studies indicate that light ions are the most probable particles for generating eye flashes (10). The measured rate of ions in the eye produced an average rate of 5 × 10^−2^ eye flashes per minute (20 in about 420 min of observation).

### Cataract

#### Spaceflight-Based Studies

Cataract risk from space radiation seems linear without a threshold caused by genetic damage leading to aberrant cellular differentiation of lens epithelial cells (11, 12). However, questions on the definition of clinical significance and the progression of cataracts with time must still be addressed for risk assessment.

The systematic investigation of lens opacification among US astronauts studied by Cucinotta et al. (13) compares historical data for cataract incidence in the 295 astronauts participated in the Longitudinal Study of Astronaut Health (LSAH) by NASA and individual occupational radiation exposure data. A dose-effect threshold of about 8 mSv, based on epidemiological [corrected] data link an increased risk of cataracts for astronauts with higher lens doses (>8 mSv) of space radiation relative to other astronauts with lower lens doses (<8 mSv). These results remain preliminary because of subjective scoring methods and suggest that relatively low doses of space radiation may predispose crew to an increased incidence and early appearance of cataracts (13).

The NASA Study of Cataract in Astronauts (NASCA) (14, 15) is a 5-year longitudinal study of the effect of space radiation exposure on the severity/progression and risk of lens opacity. The study included 171 consenting astronauts who flew at least one mission in space, and a comparison group made up of three components: (a) 53 astronauts who had not flown in space, (b) 95 military aircrew personnel, and (c) 99 non-aircrew ground-based comparison subjects. Continuous measures of nuclear, cortical, and posterior subcapsular lens opacities were derived from digitized images and were collected for assessing demographic characteristics, medical history, and habits. The variability and median of cortical cataracts were significantly higher for exposed astronauts than for non-exposed astronauts and comparison subjects with similar ages (*P* = 0.015). Cross-sectional data analysis revealed a small deleterious effect of space radiation for cortical and possibly posterior subcapsular cataract lens opacities. These results suggest increased cataract risks at smaller radiation doses than have been reported previously.

Preliminary analyses of 5 years of data with an average of 3.8 exams per subject found no relationship between radiation exposure and progression rates for posterior subcapsular cataracts and nuclear cataracts, e.g., the estimated median progression rate from space radiation being 0.25 ± 0.13% lens area/Sv/year (*P* = 0.062).

### Central Nervous System

#### Ground/Simulation-Based Study

Possible CNS risks during a space mission include cognitive function (e.g., detriments in short-term memory, reduced motor function, and behavioral changes), while late CNS risks comprise neurological disorders, such as premature aging and Alzheimer's disease or other dementia.

The risks of CNS are of concern for long-term exploration missions to Mars or other destinations, while the possible observation of CNS effects in astronauts participating in the past NASA missions is highly unlikely because in low earth orbit (LEO), the astronauts are partially protected by the magnetic field of the Earth, the lengths of past missions which are relatively short, and the small population size of astronauts (16).

The doses used in experimental studies have been much higher than the annual GCRs dose (~0.1Gy/y at solar maximum and ~0.2Gy/y at solar minimum with <50% from high charge and energy particles). Several studies have been conducted to simulate the GCR radiation using heavy-ion or proton beams to provide evidence for the CNS health risk for missions outside of LEO. Britten et al. (17) have shown that doses as low as 20 cGy of simulated GCR radiation (1 GeV/u ^56^Fe particles) can significantly impair learning and memory in a rodent model, while Hienz et al. (18) demonstrated that proton radiation caused marked neurocognitive deficits at doses as low as 25 cGy.

Radiosensitive animals exhibited significant changes in proteins associated with dopamine receptors and transporters in the brain at mission-relevant doses and dose rates. These results indicate that susceptibility should be considered in dose-effect models predicting the radiation-induced CNS changes (19). Further investigation is still mandatory to elucidate the impact of dopamine changes as a predictor for the CNS morbidity of the astronauts. CNS effects depend on multiple mechanisms leading to synapse changes (20), among other effects. The average lifetime of synapses varies in different brain regions and depends on the exposure time. In addition, the microgravity effects are also to be considered.

Space travel may cause cognitive detriments associated with changes in neuron morphology and plasticity. Observations in mice revealed a dependence on radiation quality and absorbed dose, suggesting that microscopic energy deposition plays an important role. Simplified 3D neuron models with properties equivalent to realistic neuron morphology have been developed using GEANT4 to describe the effect observed in rats after a dose from 0.1 to 2 Gy delivered to the hippocampus (21). Of note, the changes to synapses are one aspect to be considered. The papers (22, 23) provide more detailed neuron morphology and track structures.

Radiation-induced impairment of neurogenesis is a concern due to its reported association with cognitive detriments after exposure to low doses of high charge and energy particles. The possible risks to astronauts chronically exposed to space radiation could prevent astronauts from performing complex executive functions (17, 24, 25) which are to be deeply investigated after radiotherapy for brain cancers.

Cacao E and Cucinotta FA in 2016 (26) reported a predictive mathematical model of radiation-induced changes to neurogenesis for various radiation types after acute or fractionated irradiation, extending a mouse model of impaired neurogenesis in the hippocampal dentate gyrus after exposure to low-LET radiation to heavy ion irradiation. Heavy-ion irradiation leads to poor or no recovery from impaired neurogenesis at doses as low as 0.5 Gy in mice.

More recently, the first quantitative meta-analysis of the dose-response for proton and heavy-ion rodent studies has been published based on the widely used novel object recognition test, which estimates detriments in recognition or object memory (27). The log-normal model predicts a heavy-ion dose threshold of ~0.01 Gy for novel object recognition-related cognitive detriments.

### Cardiovascular Disease

#### Spaceflight-Based Studies

Based on a group of 84 flight astronauts, Delp et al. (28) found no differences in CVD mortality rate between non-flight (9%) and LEO (11%) astronauts, while the CDV risk reported among Apollo lunar astronauts (43%) was 4–5 times higher than in non-flight and LEO astronauts. Unfortunately, Delp et al. (28) did not consider the participation of the Apollo lunar mission crew in other missions, radiation doses, experimental protocols using radioisotopes, and time in space under microgravity conditions. Moreover, the incomplete collection of death certificates was available for only 49%, with the remaining information from newspaper and journal articles (29) raised severe doubt on the above conclusions.

CVD from low-dose radiation exposure represents an important issue for space missions (30) and radiotherapy as experienced in an ever-growing number of cancer survivors (31). There is no demonstrated relationship between CVD risk and low-dose cardiac exposures after a space mission, likely due to the statistical limitations of cohorts of astronauts (30) preselected among health subjects with appropriate life habits. However, associations between CVD and whole-body doses of <1Gy among atomic bomb survivors and the experience from radiotherapy are of potential clinical importance and provide a foundation for assessing astronaut health.

#### Ground/Simulation-Based Study

Radiotherapy and recent epidemiological studies have suggested that an increased risk of CVD may also arise from low-level exposure (32). The study of CVD mortality from 1950 to 2003 among the Japanese survivors of the atomic bombings of Hiroshima and Nagasaki (33), showing an increased CVD-related death risk at low doses, raised the attention of the scientific community.

Additional information relied on groups of workers exposed to ionizing radiation. The latest study by Azizova et al. (34) examined hypertension incidence (based on 8,425 cases) in Mayak (nuclear installation in the Southern Urals of Russia) workers concerning external radiation and internal plutonium. They found a significantly positive CVD incidence for external exposure, the excess relative risk (ERR) per sievert of external radiation dose being 0.13 (95% CI, 0.08–0.19), but not for plutonium exposure.

The International Nuclear Workers Study (INWORKS) has recently investigated CVD mortality among over 300,000 workers from the United States, United Kingdom, and France, reporting a statistically significant increase of ERR per sievert being 0.22 (90% CI, 0.08–0.37), based on the cumulative recorded occupational dose received from external photons (35).

However, the authors highlighted that the significant heterogeneities of workers preclude a reliable interpretation of the CVD ERR results. A 2008 study done by McGeoghegan et al. (36) on a subgroup of workers (i.e., previously operating in the facilities of British Nuclear Fuels plc.) included in the previously cited study reported the same concerns about the reliability of the CVD ERR result.

A study of CVD incidence from 1986 to 2012 using the data derived from the medical examinations of 53,772 Russian liquidators working in the Chernobyl zone during the first year after the accident found highly significant ERR per sievert estimates for the incidence of CVDs (37, 38).

At present, there is an indicative evidence for a link. However, overall, the findings at low-dose exposure are not yet persuasive due to the possible influence of major nonradiation risk factors (concomitant exposure to organic solvents and acids) on the reported associations and possible misclassification of cause of death and various potential selection effects (32, 39).

### Cancer

#### Spaceflight-Based Studies

About 3% risk of exposure-induced death is generally used as a basis for setting age- and gender-specific dose limits for astronauts based on the National Council on Radiation Protection and Measurements (NCRP) report no.132 (40). After adjusting US cancer rates to remove smoking effects, radiation risks for lung and other cancers, the radiation mortality risks for never-smokers were reduced compared to the average US population by more than 20% and 50% in the mixture model and multiplicative transfer model, respectively (41).

#### Ground/Simulation-Based Study

Cancer is a stochastic risk, and for this reason may occur even at shallow doses (defined as doses <100 mGy) which is currently estimated using the Linear-No Threshold model (LNT) according to the United Nations Scientific Committee on the Effects of Atomic Radiation (UNSCEAR) (42), the International Commission on Radiological Protection (ICRP) recommendations (43), and the NCRP commentary (44). However, the debate on the accuracy of the LNT model is still open (45–47). Furthermore, compared to X-rays, multicellular models of tumors and normal tissue due to carbon ions have also been investigated (48).

Of note, the uncertainties of cancer risk predictions to exposure to GCRs have been described within the linear-additivity model using the approach of Monte Carlo sampling from subjective error distributions. One of the sources of uncertainties is related to the behavior of quality factors (QFs) of the particles constituting the GCR at low doses. This issue represents the central gap of knowledge to quantify the overall uncertainty in risk projections. The function of particle type or charge number and energy of QFs have been intensely investigated using track structure models (41, 49). Moreover, the QF extrapolation to low dose has been verified using the sizeable radiation-induced cancer rates from the UNSCEAR (2008). In addition, the overall probability distribution functions of the NASA QF function for solid cancers and leukemia vs. kinetic energy for iron particles have been recently reviewed (50).

### Biomarkers and Chromosomal Aberrations

#### Spaceflight-Based Studies

High-LET radiation is more efficient in producing complex-type chromosome exchanges than sparsely ionizing radiation, and this can potentially be used as a biomarker of radiation quality. Chromosomal aberrations in blood samples of astronauts increase with absorbed doses (51).

The blood lymphocytes of the astronauts were analyzed before and after 3–4 months long duration missions to investigate the complex chromosome exchanges (51). The pooled data for metaphase and premature chromosome condensation analysis for all the four ISS crewmembers revealed 6 complex exchanges preflight in a total of 24,136 cells analyzed, and 12 complex exchanges were detected in 26,065 cells collected after the flight. Chromosome aberrations in the lymphocytes of the crewmembers before and after long-duration permanence on the Mir space station were measured in metaphase cells. The total number of complex exchanges detected was very low; a total of 8 complex exchanges were detected preflight in the 20,910 cells analyzed from all crewmembers combined. After flight, 20 complex exchanges were detected in a total of 30,078 cells from all the astronauts (51).

Similarly, Yang et al. (52) showed that the frequency of chromosomal aberrations increased significantly in postflight samples compared to the samples drawn before the flight and that the frequency of sister chromatid exchanges (SCEs) was similar for both pre- and postflight samples.

Further, they estimated a relative biological effectiveness (RBE) of 2.8 for the Mir-18 mission space radiation environment. To perform the RBE, they calculated the ratio between the estimated equivalent dose from chromosomal aberrations (14.75 cSv) and the measured physical absorbed dose (5.2 cGy). The dose equivalent was derived from a dose-response relationship based on the blood samples of preflight astronauts irradiated at various doses using gamma rays.

Similarly, Cucinotta et al. (53) showed that the chromosome aberration of cosmonauts receiving doses in space ranging from about 0.5 to 15 cGy were in most of the cases 2–5 times higher than the unexposed control samples. The estimated frequency of dicentric aberrations in lymphocytes was in good agreement with the observation in MIR-18 crew members. Moreover, the yield of chromosome aberrations increased after flight for five of the NASA/MIR crew members while two decreases over time, reaching the unirradiated values (i.e., the baseline values observed before the flight) (54). This behavior suggests a non-additive or even infra-additive effect, supporting that a radio-adaptive response could occur (7, 55). The bio-dosimetry based on the dicentric chromosome aberration analysis has been developed and validated (56–58) and represents a fast and reliable tool for dosimetry assessment of populations exposed to radiological incidents for triage purposes.

Two biological response models were compared to the Mir biodosimetry for chromosome aberration in lymphocyte cells; a track-structure model and the linear-quadratic model with LET-dependent weighting coefficients. Both models are in reasonable agreement with data for aberrations in the lymphocytes of Mir crew members. Of note, the difference in the models is the increased effectiveness predicted by the track model for low charge and energy ions with LET near 10 keV/micrometres, indicating that aluminum shielding, although providing necessary mitigation of the effects of trapped radiation, provides no protective effect from the GCR in LEO (53). No significant increase was observed in the yields of chromosome exchanges in the peripheral lymphocytes of astronauts increased after long-duration missions, indicating that the clearance of aberrations from the blood lymphocytes is negligible up to 240 days after the flight (54).

Gao et al. (59) reported that enhanced radiosensitivity recruits more gene and miRNA involved in DNA damage response under space radiation condition, and the microgravity further enhanced the DNA damage response on the transcriptional level. Similarly, Kaur et al. (60) reported that changes in neutrophil functions are affected by factors associated with space flight, and this relationship may depend on mission duration. Moreover, decreased non-major histocompatibility complex (MHC) restricted killer cell cytotoxicity has also been reported in astronauts after spaceflight (61).

#### Ground/Simulation-Based Study

Some of the proposed biomarkers to predict the risk of carcinogenesis include complex clustered DNA damage, persistent DNA repair foci, reactive oxygen species, chromosome aberrations, and inflammation. Other biomarkers discussed, often assayed for a longer period of postexposure, include mutations and telomere length changes (62).

Experiments performed at the NASA Space Radiation Laboratory revealed that heavy ions induce expression of the TGF-β1 isoform, which can modulate late post-radiation changes and increase the risk of tumor development and metastasis even when cells were irradiated with doses as low as 0.1 Gy (63). Further studies are needed to determine whether the chronic exposures received in space may potentiate this process in astronauts, leading to increased cancer risk.

Proteins, microRNAs (miRNAs), and transfer ribonucleic acid (tRNA)-derived fragments in serum showed great potential as early biomarkers of exposure to energetic heavy ions and might be helpful in dose reconstruction and risk assessment of heavy-ion exposure in deep space exploration (64). These biomarkers increase or decrease with the increase of the dose in the range of 10–50 cGy.

Finally, in rats irradiated with 60 cGy using 1 GeV ^56^Fe-particle, radiation impacts on hippocampal glutamatergic neurotransmissions at 3 and 6 months after exposure, which might play a critical role in learning and memory, likely causing neurocognitive impairment (65).

### Others Risks

Potential induction of mucositis in astronauts after long-term exposure to high LET/high energy particles (such as carbon ions) during extended space flights has been described as related effects (48). The effect in terms of cell density/compactness, double-strand breaks, and induction of NFkB or interleukins has been investigated using doses ranging from 2 to 10 Gy (66). Activation of the transcription factor NFκB, carbon ion, and X-rays induced the activation of NFκB in the mucosa model. Increased secretion of pro-inflammatory cytokines and chemokines is involved in initiating radiation-induced oral exposure mucositis and in linking inflammation to cancer development and progression. Again, the different qualities of radiation appear to affect mucosa cultures in different ways following different kinetics. X-rays induced an early activation of NFκB already 4 h after treatment, which returned to control levels at 24 h after treatment, while heavy-ion-induced effects reached their maximum of 48 h after treatment (66).

The human skin is exposed in every external radiation scenario, making epithelial tissue an ideal model to study radiation-induced effects, from *in vitro* 3D human organotypic skin tissue model to low doses of high LET oxygen (O), silicon (Si), and iron (Fe) ions to investigate the integrity of the barrier function of the skin, which was maintained at various particles and doses (67).

## Discussion

### Open Issues

For years, astronauts have been exposed to space radiation comprised of high-energy protons and heavy ions, and secondary particles produced in collisions with spacecraft and tissue. Unfortunately, significant uncertainties exist in projecting risks of late effects from space radiation, such as cancer and cataracts due to the paucity/corrected epidemiological data and levels of absorbed doses.

Interactions of the GCRs with the spacecraft hull will significantly impact the radiation exposure of astronauts. Charged particles traversing the hull or “shielding” of the ship will incur nuclear interactions that depend on the composition and thickness of the hull material. These interactions will result in fragmentation products and particles of reduced energy but higher linear energy transfer (LET) that contribute to the radiation dose within the spacecraft. The average radiation dose for the seven deceased Apollo crew was 0.59 ± 0.15 cGy (range 0.18–1.14 cGy). Using similar assumptions, astronauts in LEO would receive 50–100 mSv over a 6–12 month stay, of which the GCR would account for approximately two-thirds of this total dose. Thus, given their mean mission duration of 15.6 days, the deceased LEO astronauts would receive ~0.29 cGy, a GCR dose similar to the Apollo lunar astronauts. The estimated dose for the shortest round-trip to Mars would be in the order of >0.6 Sv (4). This value is close to, or even above, the dose limits proposed by NASA for the entire career of an astronaut (68).

In addition, radiation risk assessment during long-term space flights has an extremely high level of uncertainties due to the space-radiation environment, the solar magnetic field activity, and the presence of shielding with different capabilities of reducing the incident radiation, thus producing heterogeneous secondary radiation particles. Early estimates of the uncertainty on cancer mortality risk due to space radiation ranged from 400 to 1,500%, with more precise estimates showing uncertainties at the 95% confidence level of 4-fold times of the point projection (69).

### Eye Flashes

Evidence shows that, at least in part, the eye flashes seen by astronauts are correlated with charged particles traversing the retina, but further studies of the role of the flux of all the GCR particles need to be investigated. Eye flashes have been the first phenomenon suggesting the possible damage to the central nervous system of the astronauts.

### Cataract

Cataract risks for astronauts have been reported at doses (8 mSv) lower than ones proposed in the European Directive 53/2013 (15–20 mSv) for workers (70–72). A decreased dose-effect threshold has also been reported for occupational exposure (74). Indeed, the current framework of radiological protection of occupational exposed medical workers reduced the eye-lens equivalent dose from 150 to 15–20 mSv per year (72). The results from systematic investigations of lens opacification based on subjective and no-standardized lens evaluation techniques might represent a limit for the risk prediction, mainly considering that NASA is planning prolonged human-crewed space missions to Moon and Mars. The NASA-funded NASCA (14, 15) study will provide new data for estimating the lens opacification in astronauts using standardized and validated objective techniques. Preliminary data did not reveal any relationship between radiation exposure and progression rates for posterior subcapsular and nuclear cataracts.

However, longer follow-up may be needed to understand better regarding the impact of space radiation on cataract progression rates and characterize visual acuity changes.

### Central Nervous System

Space radiobiology studies of CNS effects using particle accelerators simulating space radiation and experimental models attempt to assess the CNS risk relevance relative to doses, dose-rates, and radiation quality expected on a Mars mission (16). However, the definition of clinically significant CNS risks for long-term exploration missions must be fully understood because the doses to the hippocampus of astronauts are under 0.1–0.2 Gy, while in radiotherapy, the mean and maximal doses to the hippocampus are under 10 and 17 Gy, respectively (73) with relevant radiation-induced neurocognitive impairment. CNS and CVDs may affect the health of the astronauts, although the uncertainty of these radiation-induced effects is even higher than cancer induction (44).

One of the most promising ways to prevent and mitigate the acute effects of CNS and the neurocognitive impairment during long-term spaceflight is based on the use of substances (e.g., Dammarane Sapogenins) (75). These and other possible strategies (76) are not yet included in the actual predictive models.

### Cardiovascular Disease

Cardiovascular disease due to the ionizing radiation is of paramount interest for radiotherapy treatment being still one of nowadays the most critical side effects of the treatment, nevertheless the high target conformal capability of modern accelerators. CVD depends on the heart and the lung doses (77) and pretreatment hypertensive heart disease (78).

In spaceflight studies, the correlation between CVD risk and absorbed doses is negative, while in ground-based studies, a relationship between CVD risk and low-level exposure to ionizing radiation is reported (32). One of the most critical uncertainty sources is the limited number of subjects involved in the space missions and the number of astronauts/crews with acute or late effects. This aspect leads to limited statistical power (<6%) for cardiovascular and mortalities (29). Due to the low power, further adjustments for other time-related parameters, such as age at first exposure and latency time were not considered, although these factors could change the risk of damage manifestation. NASA uses a 3% risk of exposure-induced death at the upper 95% CI as a basis for setting age- and gender-specific dose limits for astronauts (79). The actual general population of dose-effect models could be too cautionary, being astronauts preselected for many factors, including cardiovascular performance and vision, lowers risks of cancer, and circulatory and pulmonary diseases (3). Since astronauts are considered as healthy and never-smokers (NS) subjects, the expected cancer risks are 20% and 30% lower for males and females, respectively, for NS compared to the average US population (68). On the other hand, different space missions and irradiation conditions allow for investigating the dose-effect relationship in a wide range of absorbed doses and microgravity conditions. Microgravity and ionizing radiations alter the gene sets when considered separately, while they did not alter the gene sets when used in combination. These indicate a complex interaction between these factors (80).

In conclusion, a comprehensive CVD risk prediction model has not yet been achieved. Further investigation is strongly recommended before long-term exploration of space missions.

### Cancer

Galactic cosmic ray spectrum can induce cancer, cognitive deficits, changes associated with premature ageing, and degenerative effects in many organs. Most epidemiologic data results from the astronaut cohort are from exposures incurred on missions during the Space Shuttle era, where <100 mSv was accumulated by an astronaut. Nevertheless, the nominal mission length for astronauts has increased to at least 6 months in duration with exposures of 1 mSv to 1.5 mSv per day, depending on the phase of the solar cycle, the number of spacewalks performed, and the level of solar activity (81).

Even with increasing mission length and radiation exposures, it is noteworthy that no astronaut has been diagnosed with cancer attributable to space radiation to date. Although the sample size is small, follow-up times for significant exposures are limited, and cancer latency periods are from years to decades. Epidemiology studies from human exposures to gamma radiation may help predict the cancer risks attributed to GCR, but further work is needed to validate these findings. Age- and gender-specific dose limits based on incidence-based risk transfer for NS are used for a more accurate estimation of cancer risk. Gaining knowledge to improve transfer models, which entails knowledge of cancer initiation and promotion effects, could significantly reduce uncertainties in risk projections (68).

In addition, the uncertainties in estimating the risks for late effects (including cancer) from space radiation exposures arise from the variability and complexity of the radiation fields due to multiple interactions with the vehicular spacecraft or human tissues. Moreover, the limited radiobiology data using high energy and high LET particles increase the uncertainty of the radiation quality and the expected dose-rate effects (82, 83). In addition, estimation of the biological risks from space radiation remains a complex problem because of the many radiation types, including protons, heavy ions, and secondary neutrons, with few epidemiology studies for these radiation types (84). In contrast to conventional dosimetric methods (85), the biophysical description of heavy particle tracks has been addressed in the context of the interpretation of both space radiation dosimetry and radiobiology data to provide insights into new approaches to these problems.

Modern instrumentation and detectors operating in space, built for astroparticle measurements (86), allows for the estimation of GCR properties and absorbed dose with a greater accuracy, thanks to the recent availability of the Alpha Magnetic Spectrometer (AMS) detector (87–91), installed on the International Space Station (ISS), that measures charged components of cosmic rays since 2011 and is approved to be operative for all the life cycle of the ISS. *Ad hoc* Monte Carlo calculation tools (92) might validate and better estimate dose-effects relationships. This aspect could be relevant also for the improvement of countermeasure, including shielding evaluation and dosimetry of a specific astronaut irradiation condition. In this concern, a study from NASA (93) outlined greater effectiveness of polyethene compared to aluminum shielding in terms of annual dose equivalent resulting from the application of various Monte Carlo transport codes and the NASA-developed deterministic code High Z and Energy TRaNsport (HZETRN), based on solutions to the Boltzmann transport equation.

### Biomarkers

A chromosomal aberration has been mainly investigated in both space radiobiology (SPRB) and radiotherapy studies. Ionizing radiation produces a significant effect in increasing chromosomal aberrations and chromosome break, and production of dicentric and ring. For this reason, chromosome gaps are very sensitive and act as helpful biomarkers to predict radiation-induced acute and late effects (94–96).

Robust predictive models are essential to managing the risk of radiation-induced carcinogenesis. It is critical to identify early sensitive and late biomarkers that can unravel how radiation-induced cellular stress alters the risk of carcinogenesis and improves the modeling of individual risk of cancer or other long-term health consequences of exposure (62).

Study on the biological effects after exposure to high LET particles used for radionuclide therapy might further contribute to ground simulation studies and to fully understand the biological effects on radiation-induced chromosome damage in peripheral blood lymphocytes (97).

Auspicious preliminary results have shown that blood cytokine levels, and in general, the alteration of immune system parameters can be considered biomarkers of low doses of radiation exposure.

The identification of predictive biomarkers to determine both the received radiation dose (biodosimetry), as well as the radiosensitivity of individuals, may be an essential aspect for future crew selection (98).

At the state of the art, few models have a reliable and accurate estimation of the dose-effects correlations due to the complexities of the flux of GCR particles and their interactions with the human tissues. Data from radiotherapy might help to improve the risk models for space radiation (99) as for radiological or nuclear attacks due to precise knowledge of absorbed dose and objective determination of effects (100).

Space radiation and microgravity are recognized as primary and inevitable risk factors for humans traveling in space, but the reports regarding their synergistic effects remain inconclusive, and various studies highlight differences in the environmental conditions and intrinsic biological sensitivity (59–61).

The remarkable progress made in cancer research during the last decade indicated that low=dose radiation could lead to various alterations in immune system parameters, including natural killer cell activation modulation of blood cytokine levels, which plays a crucial role in cancer development (101–104) as well as in cancer control (105). This issue needs to be further explored for long-term missions.

The expected absorbed dose range to oral cavity (2–10 Gy) for astronauts is broadly lower than the threshold for the induction of oral mucositis reported for Grade 2 or more toxicity using carbon ion therapy (i.e., 43–54Gy RBE-corrected (106)) or cumulative doses of 32–42 Gy (107, 108) using photon therapy. In addition, in-flight experimentations on intestinal microbiota showed a significant change without alteration of mucosal integrity (109). These data first reinforce the critical need for further studies exploring the impact of spaceflight on intestinal microbiota to optimize long-term space travel conditions.

### Strategies for the Improvement of the Models

Further studies are mandatory to guide the development toward novel medical applications and to protect the astronauts during space exploration.

For this reason, the improvements for health hazards related to space exploration are a unique opportunity for the safe conduction of space missions. The first ground-based GCR simulator of NASA (110) enables a new era in space radiobiology research due to its capability to generate a spectrum of ion beams that approximates the primary and secondary GCR field experienced at the locations of human organs within a deep-space vehicle. This facility will accelerate our understanding and mitigation of health risks faced by the astronauts.

### Ongoing Space Radiobiology Research

Several limitations have been pointed out regarding the capability of the existing accelerator-based test facility to emulate the particle fluxes of spacecraft or planetary atmosphere shielding. The introduced uncertainties are relatively small for the solid cancer risk while they are challenging to estimate for CNS or other hazards (111).

Due to the new interest in human space exploration, the European Space Agency (ESA) is currently expanding its effort in identifying all the necessary research activities to create a European Space Radiation Risk Model (ESRRM) (112) and to obtain a harmonized set of criteria for maximum allowable exposure between all the space agencies (NASA, Jaxa, etc.). The needed research areas to increase the knowledge in the field are recently identified from a team of experts from the ESA Topical Team. Among this area, the development of a new dose-effect model as part of the “missing biology for risk assessment” has a crucial role. In this context, the ESA Topical Team recommends exploring the shape of the dose-effect relationship for radiation-induced health effects and understanding the potential impact of individual susceptibility. Substantial efforts have been made to delineate biological mechanisms and health-related outcomes of low-dose radiation. These include a sizeable Low Dose research program, funded by the US Department of Energy, operated in the 2000s, and the EU funded programs, previously NOTE and DoReMi, and currently MELODI (113). Nevertheless, QFs still demand further investigation to improve the design of the radiobiological dose-effect model. An overview of available dose-effect models for SPRB has been conducted to identify the potential improvements in this expertise field.

## Conclusion

Cancer and toxicity risks remain not accurately quantified despite the technological developments and conceptual advances of space radiobiology and considerable efforts. In the latest years, significant improvements have been made in the absorbed dose-effect estimation and the construction and development of novel ground-based galactic cosmic ray simulator facilities. Technological advancements might realize the dream of human space exploration, and crewed spaceflights to explore and colonize the Moon and Mars are on the agenda of space agencies. Radiological devices or linear accelerators might help conduct *in vitro* or *in vivo ad hoc* experiments or analyze the available information from the cohort of cancer patients, thus reinforcing our knowledge on cancer and non-cancer space-radiation induced effects. Unfortunately, the number of events helpful in modeling the radiobiological effects is still limited. Consequently, functional dose-effect models/relationships and their uncertainties need further improvement, and we suggest implementing future research to increase the understanding of biological mechanisms.

## Author Contributions

AB and LS conceived the manuscript and produced the first draft. AB, SS, and LS contributed to the Pubmed search. All the authors contributed to the critical discussion on the dose-effect models for space and improved the manuscript. All the authors approved the final version of the manuscript.

## Conflict of Interest

The authors declare that the research was conducted in the absence of any commercial or financial relationships that could be construed as a potential conflict of interest.

## Publisher's Note

All claims expressed in this article are solely those of the authors and do not necessarily represent those of their affiliated organizations, or those of the publisher, the editors and the reviewers. Any product that may be evaluated in this article, or claim that may be made by its manufacturer, is not guaranteed or endorsed by the publisher.
